# Systems integration to promote the mental health of Aboriginal and Torres Strait Islander children: protocol for a community-driven continuous quality improvement approach

**DOI:** 10.1186/s12889-020-09885-x

**Published:** 2020-11-27

**Authors:** Janya McCalman, Roxanne Bainbridge, Yvonne Cadet James, Ross Bailie, Komla Tsey, Veronica Matthews, Michael Ungar, Deborah Askew, Ruth Fagan, Hannah Visser, Geoffrey Spurling, Nikki Percival, Ilse Blignault, Chris Doran

**Affiliations:** 1 Centre for Indigenous Health Equity Research, Central Queensland University, cnr Abbott and Shield St, Cairns, QLD 4870 Australia; 2 School of Health, Medical and Applied Sciences, Central Queensland University, cnr Abbott and Shield St, Cairns, QLD 4870 Australia; 3 Apunipima Cape York Health Council, 186 McCoombe St, Cairns, QLD 4870 Australia; 4 University Centre for Rural Health, The University of Sydney, 61 Uralba St, Lismore, NSW 2480 Australia; 5 The Cairns Institute, James Cook University, McGregor Rd, Cairns, QLD 4878 Australia; 6 Resilience Research Centre, Dalhousie University, 6420 Coburg Rd, Halifax, NS B3H 4R2 Canada; 7 Primary Care Clinical Unit, The University of Queensland, Brisbane, Qld 4072 Australia; 8 Southern Queensland Health Centre of Excellence in Aboriginal and Torres Strait Islander Primary Healthcare, Building 2/37 Wirraway Parade, Inala, QLD 4077 Australia; 9 Gurriny Yealamucka Health Service, Bukki Rd, Yarrabah, QLD 4871 Australia; 10 Bulgarr Ngaru Medical Aboriginal Corporation, 153 Canterbury St, Casino, NSW 2470 Australia; 11 Australian Centre for Public and Population Health Research, Faculty of Health, University of Technology Sydney, 235 Jones St, Ultimo, NSW 2007 Australia; 12 Translational Health Research Institute, University of Western Sydney, School of Medicine, Campbelltown Campus, NSW 2560 Australia

**Keywords:** Integrated care, Mental health, children’s health, Indigenous health

## Abstract

**Background:**

Systems integration to promote the mental health of Aboriginal and Torres Strait Islander children works towards developing a spectrum of effective, community-based services and supports. These services and supports are organised into a coordinated network, build meaningful partnerships with families and address their cultural and linguistic needs, to help children to function better at home, in school, in the community, and throughout life. This study is conducted in partnership with primary healthcare (PHC) and other services in three diverse Indigenous Australian communities. It entails conceptualising, co-designing, implementing, and evaluating the effectiveness of systems integration to promote the mental health and wellbeing of Indigenous school-aged children (4–17 years). This paper outlines a protocol for implementing such complex community-driven research.

**Methods/design:**

Using continuous quality improvement processes, community co-designed strategies for improved systems integration will be informed by narratives from yarning circles with Indigenous children and service providers, and quantitative data from surveys of service providers and audits of PHC client records and intersectoral systems. Agreed strategies to improve the integration of community-based services and supports will be modelled using microsimulation software, with a preferred model implemented in each community. The evaluation will investigate changes in the: 1) availability of services that are community-driven, youth-informed and culturally competent; 2) extent of collaborative service networks; 3) identification by PHC services of children’s social and emotional wellbeing concerns; and 4) ratio of children receiving services to identified need. Costs and benefits of improvements to systems integration will also be calculated.

**Discussion:**

The study will provide evidence-informed, community-driven, and tested models that can be used for implementing systems integration to promote the mental health and wellbeing of Indigenous children. It will identify the situational enablers and barriers that impact systems integration and determine the extent to which systems integration improves service availability, systems and child outcomes. Evidence for the cost effectiveness of systems-level integration will contribute to national mental health policy reform.

## Background

### Introduction

The school years of childhood (aged 4–17 years) are a particularly important time for early intervention in mental health since half of all global mental health conditions start by age 14 years and three quarters by age 24 [1, 2]. Services and supports for early intervention are variously offered by primary healthcare (PHC) services, schools, youth services, mental health services, child protection and juvenile justice services. But, as described in 14 Australian government mental health, social and emotional wellbeing (SEWB), and suicide prevention policy documents released from 2013 to 2018, the service system is complex, inefficient and fragmented [3–5]. Navigating the system is therefore a formidable challenge for service users [5, 6].

Efforts to improve the integration of mental healthcare services have cited a need for reprioritisation and reinvestment from crisis focussed to preventive services and from visiting services towards greater emphasis on community-based and community-controlled services [7–10]. The recent Queensland Mental Health Commission strategic plan, for example, stated that “a more balanced approach requires a shift towards the community as the key place where mental health …services and support are provided...” ([9] , p. 31). Important in such community-driven approaches is the integration of mental health promotion and early intervention services for children across the primary and mental healthcare, education and social services sectors [11].

Aboriginal and Torres Strait Islander Australian (hereafter respectfully termed Indigenous while acknowledging cultural and historical diversity) communities share the broad goal of providing access to effective mental health promotion and care for their children and families [12, 13]. Experiences of systemic racism, low socio-economic environments, and the historic and ongoing marginalisation of Indigenous cultures place Indigenous children at elevated risk of poor mental health [10, 14–17]. However, efforts by Indigenous community leaders to identify priorities for improvement and implement decisions to improve the effectiveness of mental healthcare services and systems have met with limited government support and resourcing for local leadership e.g. [10, 17, 18]. In one discrete Indigenous community, for example, evaluators found a complex and disjointed network of 39 distinct programs delivered by 21 providers to the community’s 330 children [18, 19]. There was little evidence of service delivery coordination or case management with federal and state government competitive and short-term funding environments compelling service providers to ‘stick to their own turf’. The overall effects of children’s programs (positive or negative) could not be determined [19]. As cited by Maddison [20] “Indigenous Affairs policy is made in a context of enormous structural inequality where [state and federal] governments hold almost all the cards”.

Based on evidence that regardless of the number of risk factors, the presence and strength of protective factors are able to reduce risk [21], Indigenous Australians have developed the strengths-based concept of SEWB to capture “understandings of themselves and their experiences as they relate to mental health” [22]. SEWB manifests in how Indigenous peoples view the world, are affected by networks of relationships and power, their histories and connections to land or ‘country’, culture, spirituality, ancestry, family and community [23–25]. However, there is currently limited evidence for how Indigenous community-driven SEWB interventions or systems-level improvements are defined or implemented [25, 26].

Recognising the need for a more systematic and community-driven approach, Indigenous PHC and other services in three diverse communities embraced an opportunity to conceptualise, co-design, implement and evaluate integrated systems to promote the mental health of Indigenous school-aged children in their communities. Indigenous guided systems integration is defined as the development of a spectrum of effective, community-based services and supports for children’s SEWB that reflect Indigenous worldviews and culture, are organised into a coordinated network, and build meaningful partnerships with families to help them to function better at home, in school, in the community, and throughout life [27]. This paper provides the protocol used to implement such complex community-driven research. Guiding this program of research, our questions are:

1. Is it feasible to conceptualise, co-design and implement systems integration that meets the desired goals of Indigenous communities and promotes the mental health and wellbeing of Indigenous children?
2. What systems improvements are identified to achieve Indigenous communities’ goals and promote children’s mental health and wellbeing?
3. Does improved community-driven integrated mental health promotion enhance the: a) availability of services that are community-driven, youth-informed and culturally competent; b) collaborative service networks to integrate systems; c) PHC-identified children’s SEWB concerns; and d) ratio of children receiving services to identified need?

### Service fragmentation

Indigenous-specific community controlled or government PHC services are responsible for providing a first contact point with the health system, including mental healthcare. The need for PHC to support children’s mental health is evidenced by National Aboriginal and Torres Strait Islander Social Survey (NATSISS) (2014–15) results that 32% of adolescents (15–19 years) reported high levels of psychological distress [15] and Western Australian surveys that showed 26% of children 4–11 years and 20.5% children 12–17 years old were at high risk of clinically significant emotional or behavioural difficulties [14]. Yet evidence from 114 PHC services suggests that only 17.4% of attending Indigenous adolescent (15–19 years) clients were screened for SEWB concerns or mental health risks [28]. NATSISS also found that more than three quarters (77%) of Indigenous adolescents with high psychological distress reported not having seen a PHC professional in the previous 12 months [15]. Furthermore, there is little evidence of the trajectories of Indigenous young people through the mental healthcare system or mental health treatment pathways developed or modified to specifically meet the needs of Indigenous adolescents [26]. Whilst PHC services are able to refer young people to youth mental health services in many urban areas, there is also evidence that Indigenous young people do not access these services commensurate with their needs [29]. Instead, the system failure to intervene early leads to a continued heavy reliance on crisis and acute services, including hospitalisation [9, 16].Hospitalisation costs $16,676 per admitted patient [30] and serious mental illness is associated with lifetime adverse outcomes.

In schools, staff (including guidance counsellors and psychologists) provide counselling, case management and referral for social, emotional, and behavioural difficulties including mental illnesses, but little is known about Indigenous students’ use of mental health services through schools [31]. Research from the United States suggests that schools may function as a de facto mental health service; a North Carolina study found that 70–80% of children who received services for a mental health problem were seen by education sector providers, and for most children receiving mental healthcare, this was the sole source of care [32]. Australian state and territory education departments recognise the role of health in students’ engagement in learning [33], and have outlined school responsibilities for both teaching about health and managing the health and wellbeing of students while they are at school. Studies have suggested that schools could play a greater role in Indigenous students’ mental (and physical) healthcare [34]. Youth services also provide a range of programs, services and facilities that promote the mental health of children, including day sports, recreation and other programs, as well as case management, therapeutic counselling, and outreach.

Despite recognition that Indigenous children are better served by investing in family support programs delivered through Indigenous organisations rather than removal and intervention, the child protection and juvenile justice sectors provide services to a vastly overrepresented Indigenous cohort (10 times and 24 times the rate for non-Indigenous children, respectively) [35, 36]. A retrospective analysis of linked data found that children reported to New South Wales protective services during early childhood were three times more likely to be diagnosed with a mental disorder during middle childhood than children without reports. Children placed in out-of-home care were five times more likely to receive such a diagnosis [37]. There is currently limited evidence about what improvements work for these children with complex needs. However, reviews suggest the need for preventive and early intervention approaches, comprehensive assessments, joined-up or wrap-around services, attachment-based interventions, placement stability and appropriately supported carers, and integrated and appropriate aftercare [38].

There is also limited evidence for interventions to integrate such fragmented intersectoral services for Indigenous children’s mental healthcare. Our scoping review of systems integration across at least two sectors to improve the mental health of Indigenous children (4–17 years) [39] found only five Australian studies [40–44]. These evaluated and/or described diverse inquiries: two explored Indigenous community and service provider perspectives of access to mental healthcare with the aim of driving service improvement [40, 43], one described a model for consultation with Indigenous stakeholders to develop a training course [44], and two described and evaluated the process and impacts of empowerment programs for Indigenous adolescents [41, 42]. Documented enablers of implementation were the involvement of community, access and cost, collaborative multidisciplinary health services, strong relationships, cultural sensitivity, and organisational and staff capacity. Outcomes across the studies included health and human services linkages and collaboration; psychosocial functioning and stress management of children; development and promotion of appropriate health policy and protocols; and family, community and organisational empowerment [39]. Although this evidence is useful, it provides limited guidance and leaves Indigenous PHC services and other service providers struggling to identify what works in planning integrated mental health service approaches [12, 25, 39].

### Theoretical framework

The federally funded systems of care approach for children’s mental healthcare improvement in the United States (US) since 1992 has elucidated a theoretical framework for systems integration. It comprises three key components that do not comprise a separate program or ‘package of activities’, but dynamic elements that interact with the extant services and communities into which a continuous quality improvement approach is introduced [45]. They are: 1) a spectrum of services and supports that interact with children and families to deliver mental health promotion and care; 2) a set of values and principles; and 3) an infrastructure, including leadership and governance, financing, health information, and workforce capacity, and networks and partnerships among agencies and with families and children [27]. These are elucidated below.

The spectrum of children’s mental health services and supports available in a community encompasses wellness promotion, early identification and support, and treatment for serious mental illness [46]. US community efforts to enhance systems integration have focussed on expanding the array of services and supports to ensure that families have choice of services across this spectrum [27]. Some US communities also implemented strategies to enhance three key values and principles within extant networks of care. These were: the need for approaches to be family-driven and youth guided to ensure that the types and mix of services provided were flexible and aligned with the strengths and needs of the child and family; community based to ensure that the locus of control and management of systems rests within community structures, processes and relationships; and culturally and linguistically competent by developing and utilising protocols, screening tools and mental healthcare pathways that were appropriately adapted to respond to children’s needs [27]. Examples of US community strategies included enhancing family-driven and youth-guided services, using evidence-informed and promising practices, improving cultural and linguistic competence and reducing racial disparities [27].

The infrastructural changes required for systems integration included considerations of leadership and governance, financing, workforce capacity, health information, and networks and partnerships among agencies and with families and children [47]. Examples of effective US governance improvements included a community locus of management and accountability, joint strategic planning and implementation, generation of policy support, and government proposals to implement systems integration requirements. Effective financing initiatives included redeploying funds from higher to lower cost services, accessing new funding streams, and block grant funding. Capacity was enhanced by staff training, technical assistance and coaching across agencies and using evidence-informed and practice-based approaches. Health information strategies included data sharing using a single client tracking system and/or shared case records, implementing or expanding the use of technology, sharing data on outcomes and economics, and using social marketing to communicate. Partnerships were cultivated through identifying strategic leaders and champions, inter-agencies and partnerships, expanding a family-centred wraparound approach, creating care management entities, improving care coordination, and adopting quality improvement and evaluation strategies [27].

This Indigenous Australian systems Integration project draws on the US systems of care theoretical framework as a guide, but will conceptualise, co-design, implement and test strategies, values, and infrastructure with and for Indigenous Australian communities. For instance, the US identified values and principles are broadly consistent with the values and principles found to underpin Indigenous Australian SEWB programs. Although these values have not been tested in Indigenous Australian communities, they are broadly consistent with the values and principles found to underpin Indigenous Australian SEWB programs. These are a family focus; Indigenous-leadership, self-determination and community governance; and a holistic approach focussed on restoration and community resilience, recovery and healing from stress and trauma, cultural responsiveness, empowerment, context specificity and interdisciplinarity and partnerships [8, 13, 25]. However, these have not been tested in systems integration initiatives within Indigenous Australian communities.

In this project, community-driven systems integration is implemented through a continuous quality improvement (CQI) process. CQI approaches have been utilised in Indigenous PHC services in Australia for more than 15 years to “involve people in planning and executing a continuous flow of improvement to provide quality health care that meets or exceeds expectations” [48–50]. Traditionally, the PHC focus has been on CQI in clinical care. CQI approaches have achieved enhanced adherence to best practice clinical guidelines, improved regularity of client attendance [51], produced a CQI workforce and appropriate health system supports, improved organisational efficiencies and increased engagement with other organisations and community members. Promising attempts have also been made to apply CQI beyond clinical care to Indigenous food security [52], health promotion [53] and the determinants of children’s health [54]. Building from this evidence, this proposed research will be the first to extend and test the value of CQI in connecting services in collaborative, participatory research to examine the dynamic, messy and interactive conditions and strategies that are needed to advance mental health systems integration [55].

### Aims

In partnership with Indigenous PHC services and linked partner services in three diverse communities, this study aims to conceptualise, co-design and evaluate community-driven systems-level integration to promote the mental health of Indigenous children. In this study, we hypothesise that in each community: 1) community-driven mental health systems integration improvements will be feasible; 2) quality improvements will increase the identification of SEWB concerns in children screened by 10% (for early identification and intervention) and improve the ratio of children receiving services to identified need by 10%; and 3) improvements will have benefits that exceed costs.

## Methods/design

This research has senior Indigenous leadership and sits at the interface of Indigenous and Western knowledge systems that is respectful of different cultural worldviews, values and practices. Indigenous participation, and community-based engagement and employment is embedded in the research design.

### The community settings

The study builds on and consolidates pre-existing research relationships with three Indigenous PHC services: two in Queensland (QLD) and one in New South Wales (NSW) (Table 1). The three services represent diversity in PHC service governance (government or community-controlled) and location (state capital city, regional town and discrete Indigenous community). Each PHC partner offers mental health services as part of their preventative health focus and approximately 30% of the regular PHC Indigenous clients are children (*n* = 3251).

**Table 1:** Settings and number of clients aged 4–17 years

PHC Collaborator	Service Type	Area Descriptor	Child clients	% children with depression
Gurriny Yealamucka Aboriginal Health Service	Community Controlled PHC	Yarrabah, northern QLD. Outer regional. Discrete community	1007	18% mod/ severe; 52% mild+^b^ [56]
Bulgarr Ngaru Medical Aboriginal Corporation	Community Controlled PHC	Casino, northern NSW. Inner regional. Integrated community	504	Not available
Southern Queensland Centre of Excellence in Aboriginal and Torres Strait Islander Primary Health Care	Government PHC	Inala, Brisbane, QLD. Major city suburb.	1740	26%^a^ [57]

^a^Inala = 26% of 15–24 year olds scored more than 9 on the adapted Patient Health Questionnaire (a-PHQ9)

^b^Gurriny = 18% of 15–24 year olds scored a-PHQ9 > 10; 52% > 5

### Research governance

Three overlapping levels of governance inform the project’s operational research management team: community health partners, a Community Youth Advisory Group and the project investigators. Community health partners from the three participating communities include representatives from the PHC services and associated service providers. Their role is to advise research implementation and provide links with appropriate services, systems and staff in each community, to champion the development of the systems integration improvements, link with project governance groups, and oversee a part-time employed community research officer. The community research officers will assist with planning, implementing and communicating the community-based project activities. The Community Youth Advisory Group comprises youth representatives (aged 17–24 years) from each participating community. It is coordinated and facilitated by an Indigenous youth empowerment organisation, Deadly Inspiring Youth Doing Good (DIYDG), in coordination with the project manager. The role of the Community Youth Advisory Group is to advise youth-guided ways to improve the mental health and wellbeing services and systems for Indigenous children in their communities. This ensures the centrality of the youth voice and their interests and needs. The project investigators comprise academic and community partners. They provide or recommend expert multi-disciplinary investigator advice, ensure ethical and cultural research principles are observed and practised, advise the research methods, and contribute to research capabilities and outputs. Each level of governance reports to an Indigenous-led project management team that comprises research and community representatives who are responsible for operational decisions.

### Study design

This research adopts a pragmatic, prospective step-wedge design [58], with a staggered implementation of the CQI approach and a 6-monthly crossover from community one (pragmatically selected) to communities two and three. A pragmatic step-wedge design allows consideration of community and organisational contextual differences, including readiness for implementation, with the sequential implementation of the core components carefully documented in each site. The step-wedge design also assures confidence in the research findings.

### Methods

**Fig. 1:**
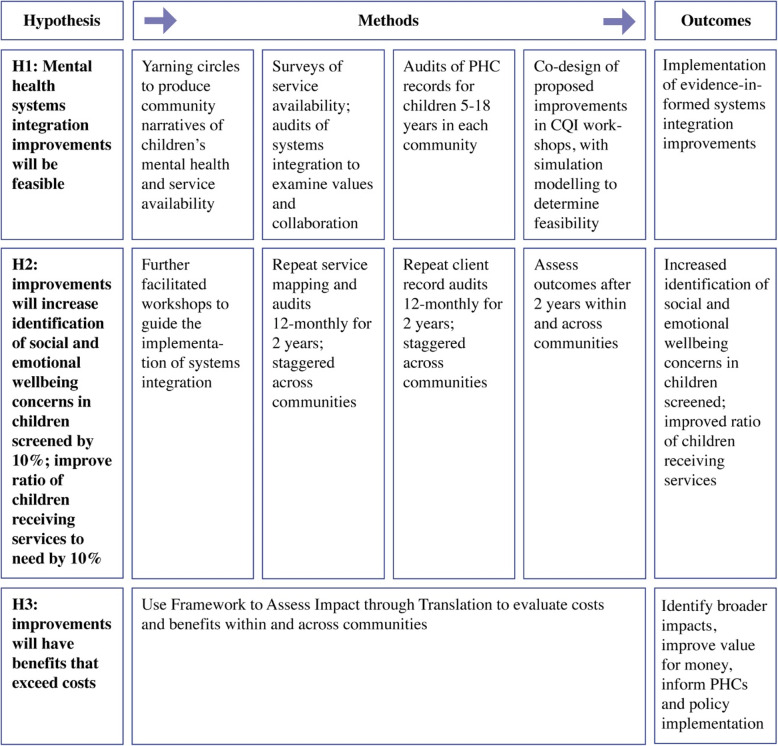
Overview of hypotheses, research methods and outcomes

In each community, a facilitated CQI approach with PHC services and linked service providers’ is structured using the feasible and flexible United Kingdom Medical Research Council framework for the development and evaluation of complex interventions [59]. Three research parts link with the three hypotheses and entail: 1) a developmental evaluation approach to inform conceptualisation and co-design of systems integration improvements; 2) an impact evaluation; and 3) an economic evaluation (Fig. 1).

### Hypothesis 1: mental health systems integration will be feasible

**Fig. 2:**
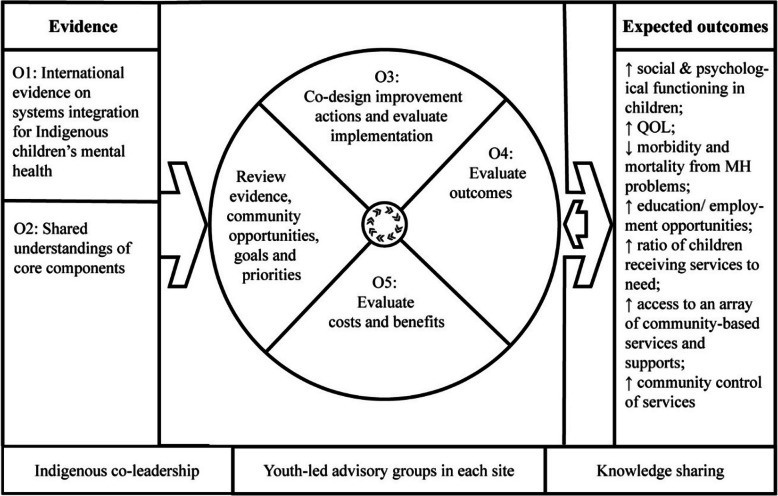
the CQI cycle

CQI workshops provide a mechanism for working with PHC services and partner organisations to co-design evidence-based and locally responsive decisions to achieve systems-level integration improvements (Fig. 2). Co-design values the Indigenous worldviews and understanding of Indigenous community service providers and children/youth. It provides opportunities to build capabilities, and develops services and initiatives that are more likely to address the issues that matter to them [60].

First, yarning circles will be held in each community to provide the perspectives of adolescents aged 12–17 years, parents/carers of children aged 4–11 years, and staff members of PHC services and partner organisations. Written consent to participate will be obtained from service providers, youth aged 16 years and over, and the parent or legal guardian of children aged 4–15 years. Discussions will focus on the characteristics of a “mentally healthy” child; supports for children’s mental health; the extent to which children seek mental health services from PHC, schools, and mental health, child welfare and/or juvenile justice services, and how agencies can better collaborate to support children’s mental health. Surveys will also be administered to provide baseline levels of service availability and the gaps in PHC, mental health, youth, school, juvenile justice and child protection services in each community. Audits of PHC records will be conducted to access data about the extent of SEWB concerns recorded for children (4–17 years). Audits of integration between community services and systems (leadership and governance financing, health information and workforce) will also be conducted to provide service providers’ perspectives on the baseline extent of collaborative service networks.

Evidence from the theoretical framework, our systematic literature review [39] and baseline findings from community yarning circles, service surveys and audits will then be analysed to pre-determine a set of core evidence-based and “testable” improvement criteria across communities. These core criteria will be presented and negotiated at community-based CQI workshops of service partners, adolescents and families. Implementation of each core element will be tailored in each community to sustainably meet the specific needs of their local situation. The community improvements will thus become co-designed flexible multi-component approaches with standardised elements and a common core assessment framework but tailored implementation and/or activities in each setting [13, 61]. CQI questions will be used to review the evidence and discuss community goals and priorities across each of the core components. Questions to be asked include: Where are we going? (Goal); What is working/not working? Who is missing out? How can we do it better? What is feasible? What improvements can we make? (Ideas); How will we know if we are making a difference? (Measurement) [62]. Grounded theory methods will be used to analyse the qualitative data from yarning circle narratives and facilitated CQI workshops [63]. A theoretical model will be developed to identify what works for whom, in what contexts, under what conditions, through which mechanisms and with what consequences.

Agreed improvement strategies will also be modelled using dynamic, discrete, event-based, microsimulation agent based modelling [64] to explore and analyse baseline systems-level integration in each community, adding the improvement decisions of each community [45, 65]. The microsimulation modelling will determine the feasibility of decisions, for understanding and clarifying the complex effects of the intersectoral community mental health service system as a whole, and predicting likely impacts and costs of proposed improvements, without disruption in real time [66, 67]. If improvement decisions are considered not feasible, the modelling will be adjusted until a desirable outcome is achieved. Once feasible, the co-designed quality improvements to systems integration will then be implemented by PHCs in each community.

### Hypothesis 2: improvements will produce impact

**Fig. 3:**
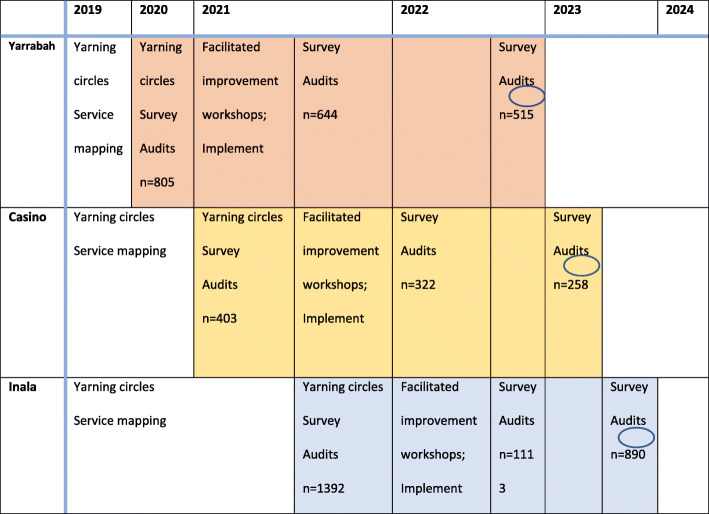
Pragmatic step-wedge design, population, research activities and indicative sample sizes

The outcomes of the co-designed improvements implemented in each PHC will be assessed by analysing changes over 2 years compared with the baseline level from each community (Fig. 3). Where possible, quantitative measures are derived from routinely collected data to measure systems integration improvement outcomes [13]. Measures will be refined based on the goals and priorities of each community and the findings from the yarning circles. The key indicators are: **1) Availability of a spectrum of community-based services that are values-based -** a survey instrument tailored to Indigenous Australian contexts from the Service System Inventory used in Canadian Circles of Care interventions [68] will be administered by researchers to community mental health services to measure the extent to which: a) Indigenous children receive mental health-related services; and b) they are community-driven, youth-informed and culturally competent. **2) Systems integration -**A modified Health Promotion Systems Assessment Tool (HPSAT) will be used to audit community-level leadership and governance, financing, workforce capacity, and health information infrastructure for systems integration, and networks and partnerships among agencies and with families and children [53]. **3) Children’s SEWB concerns** - modified child (5–11 years) health [69] and youth (12–17 years) health clinical audit tools [70] will be used to audit the PHC records of children’s attendance at the health service, reason for attendance, and discussions/ brief intervention/ advice about SEWB in the last 12 months (modified to also audit concerns identified). The youth health clinical audit tool has a specific focus on SEWB and also incorporates items for mental health-related long-term health conditions and management plan, emotional wellbeing assessment, documented concerns, recorded risk status for suicide and self-harm, actions taken, actions reviewed, referral and follow up. The actual measurement tool used may differ by PHC service and age, for example Inala and Yarrabah use the Strengths and Difficulties Questionnaire for children 5–14 years and the adapted Patient Health Questionnaire for children aged 15 years+, but important here is the identification of need regardless of the measurement tool used. **4. Ratio of children receiving services to identified need** - the ratio of children receiving mental health services to identified need will be determined by the number of Indigenous children receiving mental health-related services (survey results) compared to the number of children identified with SEWB concerns (PHC audits). The identification of need (through audits) is likely to vastly underestimate actual need but provides an indicator and base for improvement actions.

Survey and audit results will be analysed for demographic comparability between communities. Logistic regression will be completed to determine the effect of the improvement on audit scores from baseline to subsequent cycles and to assess the cycle trend. The association between the audit scores over time will be tested by fitting multilevel logistic regression models. Potential confounding variables will be explored to assess their impact on outcomes of interest, with missing data and drop-outs described. Probabilistic sensitivity and uncertainty interval analyses will be conducted to examine uncertainty around impact assessments. Cross-community comparisons will also be analysed.

### Hypothesis 3: improvements will have benefits that exceed costs

The economic evaluation will apply the Framework to Assess Impact through Translation (FAIT) to test the benefits and costs of the systems integration improvements [71]. The FAIT employs a combination of quantified metrics (a modified form of Payback) [72], economic assessment (using a social return on investment), and narratives of the process as the research translates and generates impact. To produce high impact, policy relevant research outputs, the economic cost and benefits of all quality improvements will be tested in the study [71].

## Discussion

This study contributes to the development of Indigenous children’s, family and community, PHC and government agendas for quality improvements in mental health and Indigenous health. It will achieve this by improving access to, the quality and efficiency of, and outcomes and impacts from integrated mental health systems and services for Indigenous children [73]. The documented benefits of systems integration include improvements in clinical and functional outcomes, behavioural and emotional strengths, school performance and attendance, more stable living situations; and reductions in suicide attempts, contacts with law enforcement, and reliance on inpatient care [27]. For families benefits are likely to include improvements in overall family functioning and adequate resources; reductions in strain associated with caring for a child with a mental illness, and missed days of work due to the mental health needs of the child [27]. The pragmatic benefits of the project for the three participating communities are likely to include improvements in the availability and coordination of mental health services, and evidence for advocating the reinvestment of funding from crisis focussed and visiting services to community-based and upstream (preventive) community-driven and controlled services.

This innovative and important study is being conducted during major reform of both the Australian mental health and Indigenous health systems. It will generate evidence to inform national and state efforts to improve both integration of the current fragmented state of mental health care [74], and health equity [73, 74]. It will contribute prominently to the 2016 National Mental Health Commission review recommendations for investment in an integrated prevention and early intervention approach, a focus on Indigenous Australians, support for children, use of evidence-based practice, and consistent outcome measures [74]. Expected research outcomes from this project include: 1) feasible and tested Indigenous community-driven systems integration models to promote, identify concerns and provide early support for the mental health of Indigenous children; 2) new knowledge of the extent to which systems integration improves the accessibility and quality of Indigenous children’s mental health services and their capacity to meet need; and 3) evidence of the economic impact of systems-level integration, informing opportunities for reducing the annual $60 billion national cost of mental ill-health [74].

Thus, the study fills fundamental gaps in the evidence to guide pragmatic efforts by Indigenous community PHC, Primary Health Networks and other sectors to optimally integrate systems to promote the mental health of Australia’s 263,000 Indigenous children. The considerable and growing population (33% of the Indigenous population) of Indigenous school-aged children offers undeniable strengths, including their potential as healthy, productive and engaged citizens. The study contributes to supporting and enhancing children’s wellbeing and potential by identifying new models for community-based and integrated mental health promotion and early intervention that are based on knowledge produced from each of the diverse communities. These improvements are underpinned by partnerships, engagement, collaboration, agreed values, participatory CQI, and tailored workforce training in systems integration approaches. The study also contributes to a specific policy focus on Indigenous children’s wellbeing – a necessary focus if the Australian government’s new Closing the Gap socio-economic targets are to be attained [75–77].

## Data Availability

Not applicable.

## Abbreviations

aPHQ9: Adapted Patient Health Questionnaire
CQI: Continuous quality improvement
DIYDG: Deadly Inspiring Youth Doing Good
FAIT: Framework to Assess Impact through Translation
HPSAT: Health Promotion Systems Assessment Tool
NATSISS: National Aboriginal and Torres Strait Islander Social Survey
NSW: New South Wales
PHC: Primary healthcare
QLD: Queensland
SEWB: Social and emotional wellbeing
US: United States
